# Transcriptomic classification of genetically engineered mouse models of breast cancer identifies human subtype counterparts

**DOI:** 10.1186/gb-2013-14-11-r125

**Published:** 2013-11-12

**Authors:** Adam D Pfefferle, Jason I Herschkowitz, Jerry Usary, Joshua Chuck Harrell, Benjamin T Spike, Jessica R Adams, Maria I Torres-Arzayus, Myles Brown, Sean E Egan, Geoffrey M Wahl, Jeffrey M Rosen, Charles M Perou

**Affiliations:** 1 Department of Pathology and Laboratory Medicine, University of North Carolina, Chapel Hill, NC 27599, USA; 2 Lineberger Comprehensive Cancer Center, University of North Carolina, Chapel Hill, NC 27599, USA; 3 Department of Biomedical Sciences, University at Albany, Rensselaer, NY 12144, USA; 4 Department of Genetics, University of North Carolina, Chapel Hill, NC 27599, USA; 5 Gene Expression Laboratory, Salk Institute for Biological Studies, La Jolla, CA 92130, USA; 6 Program in Developmental and Stem Cell Biology, Peter Gilgan Center for Research and Learning, The Hospital for Sick Children, Toronto, ON M5G 1X8, Canada; 7 Division of Molecular and Cellular Oncology, Department of Medical Oncology, Dana-Farber Cancer Institute and Harvard Medical School, Boston, MA 02115, USA; 8 Department of Molecular Genetics, The University of Toronto, Toronto, ON M5R 0A3, Canada; 9 Department of Molecular and Cellular Biology, Baylor College of Medicine, Houston, TX 77030, USA

## Abstract

**Background:**

Human breast cancer is a heterogeneous disease consisting of multiple molecular subtypes. Genetically engineered mouse models are a useful resource for studying mammary cancers *in vivo* under genetically controlled and immune competent conditions. Identifying murine models with conserved human tumor features will facilitate etiology determinations, highlight the effects of mutations on pathway activation, and should improve preclinical drug testing.

**Results:**

Transcriptomic profiles of 27 murine models of mammary carcinoma and normal mammary tissue were determined using gene expression microarrays. Hierarchical clustering analysis identified 17 distinct murine subtypes. Cross-species analyses using three independent human breast cancer datasets identified eight murine classes that resemble specific human breast cancer subtypes. Multiple models were associated with human basal-like tumors including TgC3(1)-*Tag*, TgWAP-*Myc* and *Trp53*^-/-^. Interestingly, the TgWAPCre-*Etv6* model mimicked the HER2-enriched subtype, a group of human tumors without a murine counterpart in previous comparative studies. Gene signature analysis identified hundreds of commonly expressed pathway signatures between linked mouse and human subtypes, highlighting potentially common genetic drivers of tumorigenesis.

**Conclusions:**

This study of murine models of breast carcinoma encompasses the largest comprehensive genomic dataset to date to identify human-to-mouse disease subtype counterparts. Our approach illustrates the value of comparisons between species to identify murine models that faithfully mimic the human condition and indicates that multiple genetically engineered mouse models are needed to represent the diversity of human breast cancers. The reported *trans*-species associations should guide model selection during preclinical study design to ensure appropriate representatives of human disease subtypes are used.

## Background

Breast cancer is the second leading cause of cancer-related deaths in American women [1]. While increased public awareness has led to earlier detection, a greater understanding of tumor biology has led to the development of many promising therapeutics [2,3]. A difficult frontier, however, has been identifying the appropriate target population for new drug(s) as not all breast cancer patients will respond to a particular therapeutic. Currently, only approximately 5% of oncology drugs that enter clinical testing are ultimately approved by the US Food and Drug Administration for use [4]. This low success rate reflects not only the difficulty of developing anticancer therapeutics, but also identifies flaws in preclinical testing methodology for selecting the most appropriate cancer patient subset for early clinical testing [5,6].

Numerous murine models of breast cancer have been created to mimic the genetic aberrations found in human tumors [7-30]. Historically, each model has been analyzed independent of other models, which complicates effective comparisons with human tumors. However, when multiple models are consolidated into a single dataset, there is increased sensitivity to detect features that are conserved with the human disease state [31,32]. Identifying murine models that faithfully mimic specific human breast cancer subtypes [33-35] is an important need for the proper interpretation of mouse model results, and thus for translating preclinical findings into effective human clinical trials [36]. To address this need, we used a transcriptomic approach to profile tumors from 27 different genetically engineered mouse models (GEMMs). We define and characterize 17 distinct murine subtypes of mammary carcinoma (referred to as classes herein to distinguish them from the human subtypes), which we compare to three human breast tumor datasets comprising over 1,700 patients to determine which GEMM classes resemble specific human breast cancer subtypes.

## Results

### Expression classes of genetically engineered mouse models

**Figure 1:**
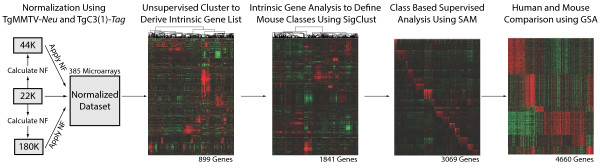
**Flowchart of murine expression data analysis.** Agilent microarrays from three different platforms were normalized and combined together to create a single murine expression dataset. Next, an unsupervised cluster analysis using variably expressed genes was performed to define a murine ‘intrinsic gene list’. Third, this intrinsic list was used as part of a supervised cluster analysis to objectively define murine subtypes/classes. Fourth, class based supervised analyses were used to define murine class specific lists (genes and pathways). Finally, supervised comparative analysis between human subtypes and mouse classes was used to identify and characterize human-mouse counterparts. NF, normalization factor. GSA, gene set analysis; SAM, Significance Analysis of Microarrays.

As the genetic aberrations of human breast cancers have been elucidated, murine models have been created to investigate the specific role that these genes/proteins have on tumor phenotype. Since our initial comparative genomics study of 14 mouse models and normal mammary tissue [31], the number of breast cancer GEMMs in our database has roughly doubled to 27 (Table 1). To compare the transcriptomic diversity of these GEMMs, global gene expression measurements from 356 unique murine tumors and 16 normal murine mammary samples were analyzed using Agilent microarrays (Table 1A, Figure 1; Table S1 in Additional file 1). Using this larger and more diverse murine dataset, a new mouse ‘intrinsic gene list’ was derived to identify genes associated with all 27 models. As expected, many of the genes from the previous intrinsic gene list were also present in the updated list. After filtering for genes found in both datasets, 76.5% (500/654) of the intrinsic probes from Herschkowitz *et al.*[31] were again included within the new intrinsic list of 1,855 probes (Table S2 in Additional file 1), which represents 1,841 genes.

**Table 1:** Summary of murine models studied

**A.**					**B.**	
**Tumor model**	**Strain**	**Promoter**	**Transgene**	**Reference**	**Primarily found in murine class(es):**	**Intramodel variation**
*Brg1*^+/-^	Mixed		*Brg1* heterozygous	[7]	Squamous-like^Ex^ (4/12); Erbb2-like^Ex^ (3/12); 3 others	Heterogeneous
Normal mammary-lactating	FVB		Normal lactating mammary tissue		Normal-like^Ex^ (2/2)	Homogeneous
*p18*^-/-^	BALB/c		*p18* homozygous null	[8]	Erbb2-like^Ex^ (5/9); Normal-like^Ex^ (2/9); Squamous-like^Ex^ (1/9)	Heterogeneous
*Pik3ca*-H1047R	FVB	MMTV	*Pik3ca* H1047R mutation overexpression	[9]	Class14^Ex^ (5/12); Squamous-like^Ex^ (5/12); 2 others	Semi-homogeneous
*Rb*^-/-^	Mixed		*Rb* homozygous null	[10]	Erbb2-like^Ex^ (4/10); Neu^Ex^ (1/10); 3 others	Heterogeneous
*Stat1*^-/-^	C57BL/6J		*Stat1* homozygous null	[11]	Stat1^Ex^ (7/7)	Homogeneous
TgMMTV-*Aib1*	FVB	MMTV	*Aib1* overexpression	[12]	Erbb2-like^Ex^ (4/9); Myc^Ex^ (2/9); 2 others	Heterogeneous
TgMMTV-*Atx*	FVB	MMTV	*Atx* overexpression	[13]	Class14^Ex^ (3/5); Squamous-like^Ex^ (1/5); 1 other	Semi-homogeneous
TgMMTV-*Fgf3*	FVB	MMTV	*Fgf3* overexpression	[14]	Erbb2-like^Ex^ (2/5); Normal-like^Ex^ (2/5); Wnt1-Late^Ex^ (1/5)	Semi-homogeneous
TgMMTV-*Hras*	FVB	MMTV	*Hras* overexpression	[15]	Neu^Ex^ (5/8); Class8^Ex^ (2/8)	Semi-homogeneous
TgMMTV-*Lpa*	FVB	MMTV	*Lpa1*, *Lpa2*, or *Lpa3* overexpression	[12]	Normal-like^Ex^ (6/15); Claudin-low^Ex^ (3/15); 3 others	Heterogeneous
TgMMTV-*Myc*	FVB	MMTV	*cMyc* overexpression	[15]	Myc^Ex^ (4/5); Class8^Ex^ (1/5)	Homogeneous
TgMMTV-*Wnt1*,i*Fgfr*	FVB	MMTV	*Wnt1* overexpression, inducible *Fgfr1* or *Fgfr2*	[16]	Wnt1-Early^Ex^ (7/12)	Homogeneous
TgWAPCre-*Etv6*	Mixed	WAP	*Etv6*-*Ntrk3* fusion gene overexpression	[17]	Erbb2-like^Ex^ (12/12)	Homogeneous
*Brca1*^+/-^, *Trp53*^+/-^, irradiated	BALB/c		*Brca1* and *Trp53* heterozygous, irradiated	[18]	p53null-Basal^Ex^ (6/7); Wnt1-Early^Ex^ (1/7)	Homogeneous
DMBA-induced	FVB		DMBA treated	[19]	Squamous-like^Ex^ (4/11); Claudin-low^Ex^ (3/11); 3 others	Heterogeneous
Normal mammary	Mixed		Normal mammary tissue		Normal-like^Ex^ (16/16)	Homogeneous
TgC3(1)-*Tag*	FVB	C3(1)	SV40 large T antigen	[20]	C3Tag^Ex^ (28/30); Claudin-low^Ex^ (2/30)	Homogeneous
TgMMTV-Cre *Brca1*^Co/Co^, *Trp53*^+/-^	C57BL/6J	MMTV	*Brca1* flox, *Trp53* heterozygous	[21]	p53null-Basal^Ex^ (4/10); Claudin-low^Ex^ (3/10); 1 other	Heterogeneous
TgMMTV-*Neu*	FVB	MMTV	Rat *Her2* overexpression	[22]	Neu^Ex^ (25/28); Normal-like^Ex^ (2/28); 1 other	Homogeneous
TgMMTV-*PyMT*	FVB	MMTV	*Py-MT* overexpression	[23]	PyMT^Ex^ (9/17); Class3^Ex^ (1/17)	Homogeneous
TgMMTV-*Wnt1*	FVB	MMTV	*Wnt1* overexpression	[24]	Wnt1-Early^Ex^ (15/25); Wnt1-Late^Ex^ (7/25); 3 others	Semi-homogeneous
TgWAP*-Int3*	FVB	WAP	*Notch4* overexpression	[25]	WapINT3^Ex^ (6/7); Class3^Ex^ (1/7)	Homogeneous
TgWAP-*Myc*	FVB	WAP	*cMyc* overexpression	[26]	Myc^Ex^ (18/21); Class8^Ex^ (3/21)	Homogeneous
TgWAP-*T121*	Mixed	WAP	*pRb*, *p107*, *p130* inactivation	[27]	Erbb2-like^Ex^ (3/6); Class3^Ex^ (2/6); Claudin-low^Ex^ (1/6)	Semi-homogeneous
TgWAP-*T121*, *Trp53*^+/-^	B6D2F1	WAP	*pRb*, *p107*, *p130* inactivation, *Trp53*het	[27]	C3Tag^Ex^ (1/1)	
TgWAP-*Tag*	C57BL/6J	WAP	SV40 large T antigen	[28]	C3Tag^Ex^ (4/4)	Homogeneous
*Trp53*^-/-^	BALB/c		*Trp53* homozygous null	[29]	p53null-Luminal^Ex^ (27/58); p53null-Basal^Ex^ (15/58); 5 others	Heterogeneous
*Trp53*^+/-^, irradiated	BALB/c		*Trp53* heterozygous, irradiated	[30]	p53null-Basal^Ex^ (4/8); Claudin-low^Ex^ (2/8); 2 others	Heterogeneous

A complete list of all GEMMs used. The bottom 15 models/normal mammary were studied by Herschkowitz *et al.*[31]. C3(1), 5' flanking region of the C3(1) component of the rat prostate steroid binding protein. MMTV, mouse mammary tumor virus. WAP, whey acidic protein.

**Figure 2:**
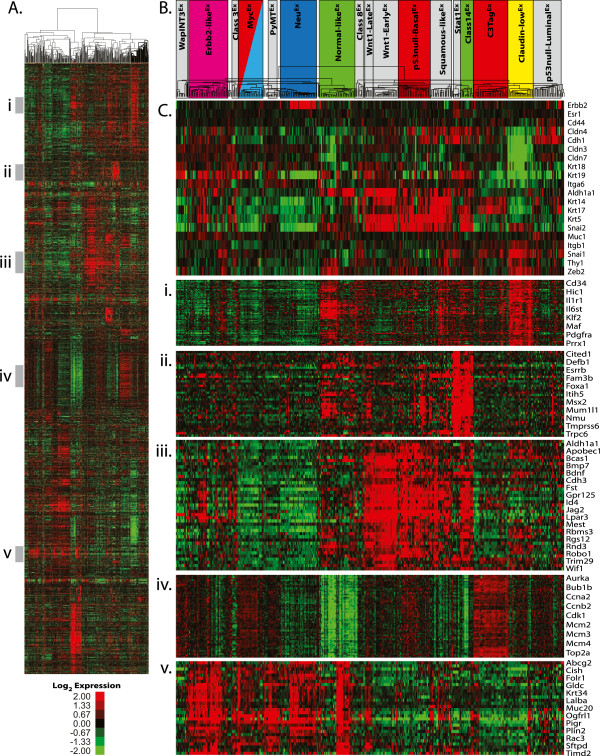
**Murine intrinsic class analysis. (A)** Supervised cluster using the newly derived murine intrinsic gene list and all murine arrays in the dataset. Roman numerals next to the gray bars correspond to the enlarged regions in parts (i) to (v). **(B)** Dendrogram of the cluster from part **(A)** with the murine classes identified by SigClust highlighted. Classes with colored boxes have been determined to be human expression-based subtype counterparts. **(C)** Breast cancer genes and individual cell lineage marker expression profiles. **(i)** Claudin-low gene cluster. **(ii)** Luminal gene cluster. **(iii)** Basal gene cluster. **(iv)** Proliferation gene cluster. **(v)** Lactating gene cluster.

To determine if new murine subtypes/classes exist in this expanded dataset, SigClust analysis [37] was performed using supervised hierarchical clustering of the 385 murine microarrays and the intrinsic 1,855 probe list (Figure 2). Murine ‘classes’ were defined as having at least five tumors with a SigClust *P*-value ≤0.01. Using these criteria, 17 murine classes were identified with 94% (363/385) of tumors being included within one of these classes (Figure 2B; Figure S1 in Additional file 2). The name for each class was determined based upon the major model contributor (for example, Myc^Ex^), the major biological feature (for example, Squamous-like^Ex^), or both (for example, p53null-Basal^Ex^), with the superscript ‘Ex’ designation used to denote that this is an expression-based class. As previously observed [31], the *Brca1*^*+/-*^*Trp53*^*+/-*^ irradiated, TgC3(1)-*Tag*, TgMMTV-*Neu*, TgWAP-*Int3*, TgWAP-*Myc*, and TgWAP-*Tag* murine models have ‘homogeneous’ gene expression patterns in this dataset; here, a model was considered ‘homogeneous’ if ≥80% of tumors from that GEMM were found within a single expression-defined class (Table 1B; Figure S2 in Additional file 2). Many of the newest models also showed homogeneous gene expression patterns, including *Stat1*^-/-^, TgMMTV-*Myc*, TgMMTV-*Wnt1/iFGFR2*, and TgWAPCre-*Etv6*.

Other models showed a ‘semi-homogeneous’ gene expression pattern, defined as ≥80% of tumors from a single GEMM being found within two classes. These included *Pik3ca*-H1047R, TgMMTV-*Atx*, TgMMTV-*Fgf3*, TgMMTV-*Hras*, TgWAP-*T121*, and TgMMTV-*Wnt1.* Interestingly, while maintaining the TgMMTV-*Wnt1* mouse colony, it was observed that there might be two types of tumors based on latency, namely early and late arising tumors. This observation was also reflected in the two TgMMTV-*Wnt1* expression classes that also differed by median tumor latency: Wnt1-Early^Ex^ (8.8 weeks) and Wnt1-Late^Ex^ (22.2 weeks) (Wilcoxon Rank Sum *P*-value <0.001). Lastly, about 40% of mouse mammary tumor virus (MMTV) driven *Wnt1* tumors have cooperative activation of fibroblast growth factor signaling [38], a phenotype that is known to decrease tumor latency [16], and consistent with this, 88% (7/8) of TgMMTV-*Wnt1/iFgfr2* tumors in our dataset were also classified as Wnt1-Early^Ex^.

The remaining models had ‘heterogeneous’ gene expression patterns, which were defined as no two classes containing at least 80% of the tumors analyzed: *Brg1*^+/-^ (five classes), DMBA-induced (five), *p18*^*-/-*^ (three), *Rb1*^*-/-*^ (five), TgMMTV-*Aib1* (four), TgMMTV-Cre *Brca*^*Co/Co*^*Trp53*^*+/-*^ (three), TgMMTV-*Lpa* (four), *Trp53*^*-/-*^ (seven), and *Trp53*^*+/-*^ irradiated (four). Similar to recent reports [32], the *Trp53*^*-/-*^ model (which is distinct from the *Trp53*^*+/-*^ irradiated model) was primarily defined by three murine classes in this analysis: p53null-luminal^Ex^ (27/58), p53null-basal^Ex^ (15/58), and Claudin-low^Ex^ (7/58).

To begin investigating the defining features of these classes, a comparison of selected cell lineage markers was performed (Figure 2C). Several mouse classes highly expressed luminal cell markers (for example, *Erbb2*, *Esr1*, *Krt18*, and/or *Krt19*), including Erbb2-like^Ex^, PyMT^Ex^, Neu^Ex^, Myc^Ex^, and Stat1^Ex^. Other classes expressed basal cell cytokeratins (for example, *Krt5*, *Krt14* and/or *Krt17*), including Wnt1-Late^Ex^, Wnt1-Early^Ex^, p53null-Basal^Ex^, Squamous-like^Ex^, Class14^Ex^, and C3Tag^Ex^. As identified previously [31], a murine Claudin-low^Ex^ class was observed to be characterized by low expression of multiple cell adhesion genes (*Cldn3*, *Cldn4*, and *Cldn7*) and high expression of epithelial-to-mesenchymal transition genes (*Snai1* and *Zeb2*), similar to the human claudin-low subtype [34].

### Comparison of murine class defining gene sets versus human tumor subtypes

**Figure 3:**
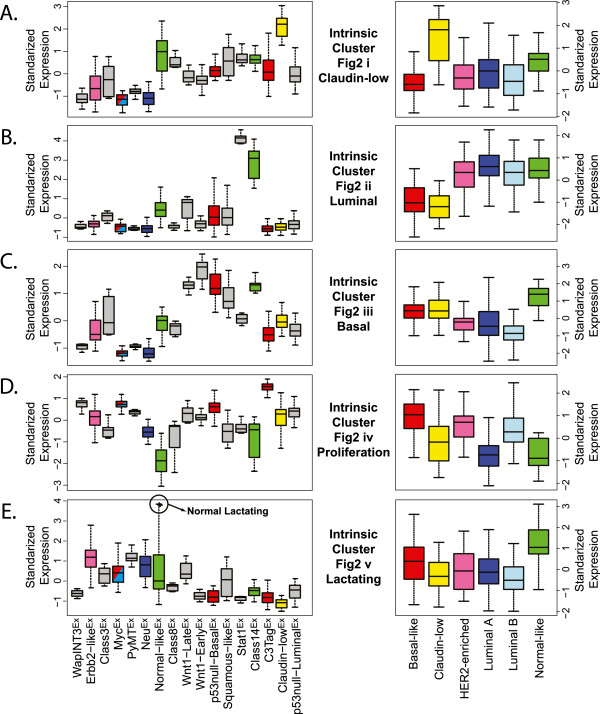
**Murine intrinsic cluster signatures according to tumor subtype.** Standardized, average expression values for the dominant individual gene clusters from Figure 2i-v are shown according to the murine classes (left panels) and the human subtypes (right panels) using the human UNC308 human breast cancer dataset. **(A)** Murine claudin-low subtype defining gene set. **(B)** Murine luminal subtype gene set. **(C)** Murine basal-like subtype gene set. **(D)** Murine proliferation-associated gene set. **(E)** Murine lactation associated gene set.

To specifically compare murine classes to human breast cancer subtype features, each murine class defining signature (Figure 2i-v) was tested for differential expression across the human subtypes using the UNC308 dataset (Figure 3A-E) [34]. For example, the high expression signature that defines the murine Claudin-low^Ex^ class (Figure 2i; including *Hic1*, *Il6st*, *Klf2*, *Maf*, *Pdgfra*, *Prrx1*, *Snai1*) was also the most highly expressed in human claudin-low tumors (Figure 3A). Figure 2ii shows genes that are highly expressed in the newly identified Stat1^Ex^ and Class14^Ex^ murine classes, which show luminal characteristics (for example, *Foxa1*, *Esrrb*) and are the most highly expressed in human luminal A tumors (Figure 3B). While most of the GEMMs in this dataset are considered estrogen receptor (ER) negative, murine models comprising these two classes (*Stat1*^-/-^ and *Pik3ca*-H1047R, respectively) were often ERα^+^[9,11], and these data suggest that they overall have a ‘luminal’ expression profile. Interestingly, these classes cluster independent of the previously defined murine luminal models, TgMMTV-*Neu* and TgMMTV-*PyMT*. Consistent with the individual cell lineage marker analysis, the Wnt1-Late^Ex^, Wnt1-Early^Ex^, p53null-Basal^Ex^, Squamous-like^Ex^, and Class14^Ex^ murine classes express a basal-like gene signature (Figure 2iii). As in human tumors, a proliferation signature (Figure 2iv) further distinguishes these murine classes, with highest expression in murine C3Tag^Ex^ and human basal-like tumors, and lowest expression in normal tissues from both species. This finding is likely due to the loss of RB1 function in both human basal-like [39,40] and TgC3(1)-*Tag* murine tumors (due to T-antigen expression). Lastly, Figure 2v highlights a gene cluster that is highly expressed in several murine classes, including Erbb2-like^Ex^, PyMT^Ex^, and Neu^Ex^; this signature was lower in normal mammary tissue, but highly expressed in the two lactating mammary samples (Figure 3E). Consistent with this observation, many of the genes in this signature are involved in alveolar function (for example, *Abcg2*, *Folr1*, and *Lalba*).

For the dual purpose of validating our new classification system and for investigating the degree of diversity in our expanded dataset, the murine classes defined here were compared to those from Herschkowitz *et al.*[31] (Figure S3 in Additional file 2). The majority of the Herschkowitz *et al.* classes had one-to-one matching counterparts to those described here; however, two previous groups (IX-WapTag and X-C3Tag) were combined into a single class in our dataset (C3Tag^Ex^). Importantly, several of the 17 murine classes defined here were not present within the 10 classes of Herschkowitz *et al.* (Erbb2-like^Ex^, Class3^Ex^, Class8^Ex^, and Stat1^Ex^), almost all of which were populated by GEMMs that were new to this study.

**Figure 4:**
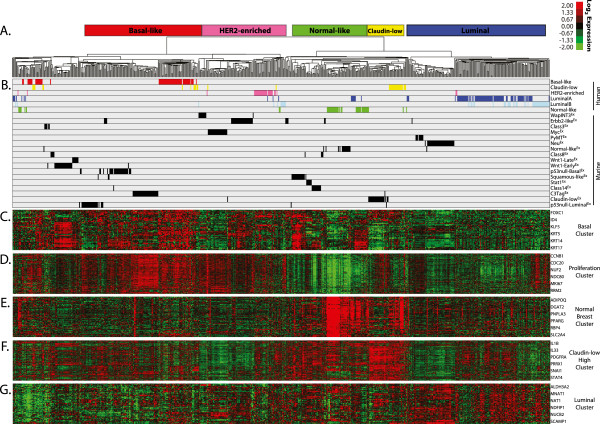
**Human and murine intrinsic co-cluster. (A)** Supervised cluster using a combined human and mouse intrinsic gene list and all murine and UNC308 human arrays. Broad tumor clusters are highlighted with names corresponding to the major human subtype(s) found within each. **(B)** Clustering location of all tumors as displayed by their human subtype or mouse class. **(C)** Basal gene cluster. **(D)** Proliferation gene cluster. **(E)** Normal breast gene cluster. **(F)** Claudin-low subtype high expression gene cluster. **(G)** Luminal gene cluster.

Given the discovery of novel murine classes, it was of great interest to determine the degree to which this expanded murine dataset might better encompass the molecular diversity of the human subtypes. To directly compare tumors across species, this mouse and the previously published UNC308 human datasets were normalized into a single expression dataset and hierarchical clustered using a combined mouse and human [41] intrinsic gene list (Figure 4). While technical differences between the two datasets (for example, different microarray platforms, different common references) may limit interspecies clustering, several across species dendrogram nodes were observed (Figure 4A). Interestingly, all major nodes contained a combination of human and mouse subtypes (Figure 4B), indicating a degree of similarity not only between specific corresponding tumor subtypes, but also globally across species. Most of the major intrinsic gene sets driving the nodes are highlighted below the dendrogram, including the basal (Figure 4C), proliferation (Figure 4D), normal breast (Figure 4E), claudin-low subtype high expression (Figure 4F), and luminal (Figure 4G) signatures. These clusters highlight the broad conserved intrinsic features between mouse and human tumors. For instance, most C3Tag^Ex^ tumors cluster with the basal-like subtype, an association that is driven in part by the high expression of the proliferation gene set [31], which is known to contain many E2F-regualted genes.

To more objectively validate the *trans*-species associations observed in Figure 4, similarity between specific human and mouse subtypes was measured using gene set analysis (GSA) (Table 2) [42]. Using this approach, a murine class was judged to be a strong human subtype counterpart if the human-to-mouse comparison was statistically significant (*P* ≤ 0.05) in at least two of the three human datasets analyzed (UNC308 [34], Combined855 [43], and TCGA547 [39]). As previously observed [31], the murine Normal-like^Ex^, C3Tag^Ex^, and Claudin-low^Ex^ classes associate with the human normal-like, basal-like, and claudin-low subtypes, respectively. The new murine class, Erbb2-like^Ex^, was associated with the human HER2-enriched subtype across all three human data sets; this human breast cancer subtype did not associate with any previously characterized murine class [31], indicating an increased ability for the current dataset to encompass more of the major human intrinsic subtypes. With this larger sample size, a link was also identified between the Myc^Ex^ class and human basal-like breast cancer, which is consistent with multiple human studies linking basal-like breast cancers with *cMYC* amplification and expression signatures [39,44]. Interestingly, a connection between the Myc^Ex^ class and human luminal B tumors was also identified, highlighting Myc activation as a potentially important etiological mechanism that is shared between these two aggressive human subtypes.

**Table 2:** Gene set analysis of murine classes and human subtypes

	**Human breast cancer subtype**																		
**Mouse class**	**Basal-like**			**Claudin-low**			**HER2-enriched**			**Luminal A**			**Luminal B**			**Normal-like**			**Predicted human counterpart**
	***P*-value**			***P*-value**			***P*-value**			***P*-value**			***P*-value**			***P*-value**			
	**U**	**C**	**T**	**U**	**C**	**T**	**U**	**C**	**T**	**U**	**C**	**T**	**U**	**C**	**T**	**U**	**C**	**T**	
**WapINT3**^**Ex**^	0.06	0.09	0.17	-	-	NA	-	-	-	-	0.44	-	0.40	0.34	0.29	-	-	-	
**Erbb2-like**^**Ex**^	0.33	0.30	0.33	-	-	NA	<1e-4*	0.01*	0.01*	0.31	-	-	0.44	0.40	0.30	-	-	-	HER2-enriched
**Class3**^**Ex**^	-	-	-	0.46	-	NA	0.41	0.17	0.38	0.31	0.28	0.34	-	-	-	0.12	0.14	0.29	
**Myc**^**Ex**^	0.02*	0.01*	0.03*	-	-	NA	0.22	0.11	0.07	-	-	-	0.06	0.01*	0.02*	-	-	-	Basal-like and Luminal B
**PyMT**^**Ex**^	0.41	0.38	-	-	-	NA	0.28	0.09	0.08	0.08	0.33	0.46	**0.02**	0.10	0.12	-	-	-	
**Neu**^**Ex**^	-	-	-	-	-	NA	0.44	0.36	0.42	<1e-4*	0.01*	**0.02**	0.10	0.36	0.43	-	-	-	Luminal A
**Normal-like**^**Ex**^	-	-	-	0.14	0.21	NA	-	-	-	-	0.07	0.11	-	-	-	<1e-4*	0.01*	5e-4*	Normal-like
**Class8**^**Ex**^	-	-	-	0.09	0.06	NA	0.48	-	-	0.40	0.46	0.11	-	-	-	0.28	0.25	0.26	
**Wnt1-Late**^**Ex**^	0.37	-	-	-	-	NA	-	-	-	0.40	0.41	0.42	-	0.46	0.40	0.15	0.01*	0.21	
**Wnt1-Early**^**Ex**^	0.29	-	-	-	-	NA	-	-	-	0.40	0.19	0.33	0.38	0.40	0.49	0.39	0.08	0.21	
**p53null-Basal**^**Ex**^	**0.04**	**0.05**	0.06	-	-	NA	-	-	0.16	-	-	-	0.48	0.29	0.20	-	-	-	Basal-like
**Squamous-like**^**Ex**^	-	**-**	0.35	0.11	0.02*	NA	0.20	-	-	-	-	-	-	-	-	0.18	0.09	0.10	
**Stat1**^**Ex**^	-	-	-	0.37	0.32	NA	0.07	-	-	0.31	0.30	0.16	-	0.48	0.41	0.38	0.39	-	
**Class14**^**Ex**^	-	-	-	0.35	0.22	NA	-	-	-	0.17	0.14	0.01*	0.45	-	0.11	0.06	<1e-4*	0.04*	Normal-like
**C3Tag**^**Ex**^	0.02*	0.02*	0.03*	0.38	-	NA	-	-	0.24	-	-	-	0.28	0.12	0.02*	**-**	-	-	Basal-like
**Claudin-low**^**Ex**^	-	-	0.38	5e-4*	<1e-4*	NA	-	-	-	-	-	0.20	-	-	0.41	**-**	-	0.17	Claudin-low
**p53null-Luminal**^**Ex**^	0.17	0.07	0.02*	-	-	NA	0.35	0.23	0.15	-	-	-	0.24	0.24	0.16	**-**	-	-	

A comparative analysis of each murine class versus each human subtype. Statistically significant observations are highlighted with an asterisk (*P* < 0.05, false discovery rate <0.1). Comparisons without a *P*-value were not found to have a positive association with each other. Abbreviation: U, UNC. C, Combined. T, TCGA. NA, not applicable.

Previously defined as a ‘luminal’ model [31], the Neu^Ex^ murine class associated with the human luminal A subtype in this newest analysis; this correlation was somewhat surprising given the lack of ERα and ERα-regulated gene expression in the murine Neu^Ex^ class, but does suggest that human luminal A tumors have many ERα-independent features. Although the murine p53null-Basal^Ex^ versus human comparisons were not significant after controlling for multiple comparisons, an almost consistent significant association was seen with human basal-like tumors (*P*-value = 0.04, 0.05, and 0.06) in all three human datasets. Lastly, Class14^Ex^ tumors were identified as a counterpart for normal-like human tumors, and of the 13 murine tumors comprising this class, 38% (5/13) are from the *Pik3ca*-H1047R model. This class clusters independent of normal mammary tissue samples (which are all classified as Normal-like^Ex^), indicating that this association is possibly not driven by contamination of normal tissue in the tumor biopsies.

### Conserved tumorigenic pathway signatures identified between human-mouse counterparts

**Figure 5:**
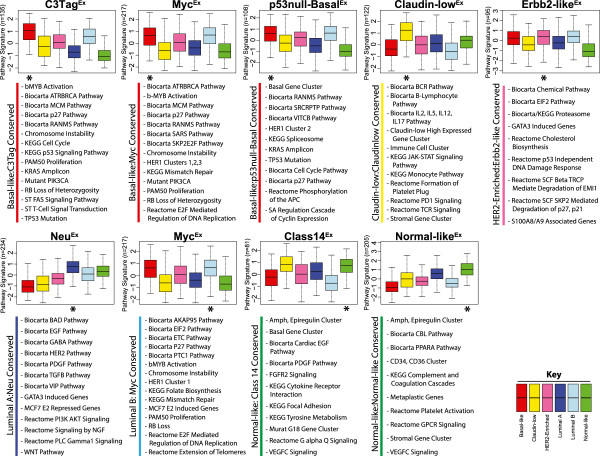
**Conserved signaling pathways between human-mouse counterparts.** A two-class SAM (class X versus all others) was used to identify pathways highly expressed in each murine class. Pathways highly expressed with a FDR of 0% were grouped together to define a ‘pathway meta-signature’ for each murine class (with the total number of pathway signatures included shown on the left axis). The standardized, average expression values of each ‘pathway meta-signature’ were calculated in the UNC308, Combined855, and TCGA547 human datasets, which are displayed as boxplots according to their intrinsic human subtype. A subset of the pathways independently identified to be highly expressed in both human-mouse counterparts (as indicated by the asterisk) for all three human datasets is displayed below each plot.

Many researchers have hypothesized that gene expression signatures may be a more robust means of utilizing gene expression data for discovery and pathway-based classification as they are composed of tens to hundreds of coordinately expressed genes. To take advantage of this approach, the median expression values for 963 publicly available pathway gene-signatures (Table S3 in Additional file 1) were calculated separately for the mouse and human datasets, and a two-class (class X versus all others) Significance Analysis of Microarrays (SAM) was used to identify pathways that were highly expressed by each class/subtype with a false discovery rate (FDR) of 0% (Tables S4-S26 in Additional file 1). To visualize pathway similarities across species, gene signatures highly expressed within each mouse class were first grouped into ‘pathway meta-signatures’, similar to the way coordinately expressed genes can be grouped into ‘gene signatures’. The average value of these ‘pathway meta-signatures’ was then calculated for each human tumor and displayed as standardized boxplots based on their human breast cancer subtype for the eight mouse classes with human counterparts (Figure 5). These boxplots allow for broad trends to be observed between the pathways highly expressed within each mouse class relative to human tumors, and in all instances, identified tens of pathway signatures that were commonly expressed across species. For instance, the average expression of the 135 pathway signatures highly expressed in C3-Tag^Ex^ tumors were also very highly expressed in human basal-like tumors (Figure 5, top left panel), consistent with the gene level analysis. While these trends are informative, it was of most importance to identify the specific pathways that were highly expressed in both mouse and their human counterparts; it is likely that these shared pathways provide etiological insight and highlight potentially important cancer driving pathways. A subset of the pathways identified as highly expressed in both human and mouse counterparts are displayed below each graph, with all across-species conserved pathways presented in Table S3 in Additional file 1.

Three murine classes overlapped with human basal-like tumors (Figure 5). One common feature between these human and mouse tumors included *Trp53* loss/mutation, which in human basal-like tumors occurs in >85% of the samples [39]. This trait was most apparent in C3-Tag^Ex^ and p53null-Basal^Ex^ murine tumors on both the genetic and the expression level. The second cardinal feature of human basal-like tumors is high proliferation, primarily resulting from retinoblastoma protein loss [39,40]. Consistent with this finding, all three basal-like mouse classes highly expressed cell cycle and/or retinoblastoma pathway-related signatures. In addition, C3Tag^Ex^ tumors were enriched for KRAS amplicon genes, b-MYB activation, mutant PIK3CA, and FAS signaling. Murine Myc^Ex^ tumors were also enriched for b-MYB activation and mutant PIK3CA signaling, in addition to a HER1-pathway signature and E2F signaling. Lastly, the p53null-Basal^Ex^ class was enriched for a SRC activation signature, a HER1-pathway signature, and the KRAS amplicon. These findings are relevant since it has been shown that human basal-like tumors also highly express the b-MYB signature [45], are often KRAS [46] and cMYC amplified [39], and show a PIK3CA-activation signature [39,47]. Thus, for human and murine basal-like cancers, both the underlying molecular genetics and their expression profiles are very similar across species.

Human and mouse claudin-low tumors also share many features, including high expression of immune cell associated genes/signatures (for example, B cell receptor, PD1, and T cell receptor signaling), which is likely due to consistently infiltrating immune cells. Both human HER2-enriched and murine Erbb2-like^Ex^ tumors highly expressed the EIF2 pathway, GATA3 induced genes, and p53 independent DNA damage response genes. Human luminal A and murine Neu^Ex^ tumors exhibited high expression levels of several tyrosine kinase-associated pathway signatures, including EGF, HER2, PDGF, TGFβ, and PIK3CA signaling. In support of this EGF/HER2 pathway finding, it was recently shown that TgMMTV-*Neu* tumors therapeutically respond to lapatinib (a dual EGFR and HER2 inhibitor) treatment [48], as would be predicted by the nature of this transgene. In addition to mimicking human basal-like tumors, the murine Myc^Ex^ class was also a counterpart for the luminal B subtype. Interestingly, many of the same pathways that were common with basal-like tumors are also shared with luminal B tumors, highlighting potentially important etiological events that are shared between these two aggressive intrinsic subtypes; these features include proliferation/retinoblastoma related pathways, increased chromosome instability, and altered DNA damage repair mechanisms.

## Discussion

Human breast cancer is a genetically complex disease consisting of well characterized molecular subtypes [33,35]. Mouse models can provide an excellent resource to study human disease, but it is essential to ensure the chosen models accurately replicate genetic alterations and overall phenotypes observed in human tumors. Thus, a number of considerations must be kept in mind when designing and/or selecting GEMMs to mimic the human disease state; these features should include intramodel tumor diversity, the degree of genetic similarity, the degree of transcriptomic similarity, and histological similarity (a topic not addressed here). By consolidating mouse models of breast carcinoma into a single dataset, this study was able to investigate the first three of these issues, in which we identified murine models for all of the major human expression subtypes.

To address intramodel tumor diversity, three types of models were identified based on hierarchical clustering analysis: ‘homogeneous’ , ‘semi-homogeneous’ , and ‘heterogeneous’. ‘Homogeneous’ GEMMs were associated with a single murine expression class and were generally created through the expression of oncogenes, possibly relying less on secondary or tertiary mutations that arise during tumor progression. These GEMMs make good experimental models because the phenotypes of individual tumors are consistent and similar. ‘Semi-homogeneous’ models, such as TgMMTV-*Wnt1*, were associated with two murine classes. We hypothesize that unknown secondary events after the initial transgene lesion determine the class fate of these developing tumors. These varying combinations of secondary lesions may cooperate with aberrant Wnt1 signaling to target different mammary cell populations, contributing to model complexity. The last type of model comprises tumors with ‘heterogeneous’ gene expression patterns (that is, models showing three or more distinct phenotypes). In contrast to ‘homogeneous’ models, the majority of the ‘heterogeneous’ models were based on disrupting the function of tumor suppressor genes. Again, we hypothesize that secondary events after the initial transgene lesion are involved in the class fate determination of these tumors. For example, the *Trp53*^*-/-*^ model shows specific DNA copy number changes associated with each expression class [32]. From an experimental perspective, special considerations (that is, phenotyping each individual tumor) must be made to account for this heterogeneity, especially when these models will be utilized for therapeutic efficacy testing.

Despite the diversity of the models tested here, we found that these mouse models collapse into distinct murine classes that recapitulate specific human subtypes on a gene expression-based level. These results are important as they allow for the identification of shared characteristics/lesions between murine and human tumors, and they direct researchers toward appropriate *in vivo* models of specific human subtypes for future experimental testing. Basal-like breast tumors are one the most aggressive subtypes of breast cancer. Herein, we find that three murine classes recapitulated human basal-like breast cancers: C3Tag^Ex^, Myc^Ex^, and p53null-Basal^Ex^. The human basal-like subtype is characterized by high proliferation [49], genomic instability [46], and expression of a c-MYC signature [39,44]. These murine classes share these hallmarks as evident by high expression of the proliferation gene cluster, cell cycle pathways, and chromosome instability gene-signatures; thus, there are clear GEMMs of human basal-like tumors that share both common genetic drivers and expression features.

Murine Claudin-low^Ex^ tumors were identified that significantly mimic the human claudin-low subtype; however, no homogeneous murine model was specific to this class/subtype. Instead, rare tumors from multiple heterogeneous models coalesced into the murine claudin-low group. As an experimental solution to this heterogeneous GEMM complication, the T11 orthotopic, transplantable syngeneic model was derived from a Claudin-low^Ex^ BALB/c *Trp53*^*-/-*^ tumor (753R), which maintains its claudin-low expression features even after multiple transplant passages [32]. This transplantable model has been used for extensive therapeutic testing [48], thus suggesting that one method of ‘capturing’ a heterogeneous model in a single state can be accomplished via the serial transplantation of a phenotypically characterized individual tumor. As in the human claudin-low subtype, *Trp53* mutation/loss was a common genetic event in mouse Claudin-low^Ex^ tumors. Similarly, both species highly express epithelial-to-mesenchymal transition related genes and inflammatory gene signatures, and have low expression of many epithelial cell adhesion genes, including E-cadherin [34].

Discovered here was the Erbb2-like^Ex^ murine class, which associated with human HER2-enriched tumors even without highly expressing the *Erbb2* gene; no mouse model from our previous studies mimicked this aggressive human tumor subtype. One homogeneous model was found within this class, namely TgWAPCre-*Etv6*. This model expresses the *Etv6*-*Ntrk3* fusion gene product, a protein that has been associated with secretory breast cancers [50]. Consistent with this, we observed that murine Erbb2-like^Ex^ tumors highly express a gene signature in common with lactating normal mammary tissue.

For the human luminal breast cancer subtypes, our previous study identified that the TgMMTV-*Neu* model represents the luminal subtypes more than it resembles HER2-enriched tumors [31]. We provide further evidence here that the murine Neu^Ex^ class specifically associates with human luminal A tumors. Conserved with humans, murine Neu^Ex^ tumors highly express several tyrosine kinase pathway-related gene-signatures, namely EGFR and HER2, which would be expected based upon the nature of the Neu/ERBB2 transgene. It has been shown that TgMMTV-*Neu* tumors regress with lapatinib treatment [48], giving credence to our approach for identifying drug targetable driver/maintenance pathways in these tumors using a computational pathway-based approach. Interestingly, only the murine Myc^Ex^ class was shown to consistently associate with luminal B tumors. Since the Myc^Ex^ class was also identified as a basal-like model, aberrant Myc activation may be a common hallmark of these two aggressive subtypes.

While our main focus was to identify human-to-mouse disease counterparts, about half of the mouse classes did not statistically associate with specific human subtypes by our broad analysis. Several of these mouse-specific classes, however, had clear basal-like tumor expression features, including WapINT3^Ex^, Wnt1-Late^Ex^, Wnt1-Early^Ex^, and Squamous-like^Ex^. Unlike the other three, the Squamous-like^Ex^ class consisted of a variety of models (for example, *Pik3ca*-H1047R, *Brg1*^+/-^, and DMBA-induced) and trended toward an association with human claudin-low tumors. Similarly, several classes had luminal expression features, highlighted by PyMT^Ex^ and Stat1^Ex^. Although the PyMT^Ex^ class had a relatively small number of samples, these tumors trended toward an association with the luminal B subtype. The Stat1^Ex^ class also had several strong luminal features, consistent with prior characterization of this model [11]. Given the expression of ERα in these *STAT1*-defecient tumors [11], the lack of an association with either the luminal A or luminal B human subtypes was unexpected.

An unanswered question concerning these human-to-mouse associations is the finding that murine classes like Erbb2-like^Ex^, and Neu^Ex^, associate with specific human subtypes despite the fact that they apparently do not show expression of one of these human subtype-defining genes (*HER2/ERBB2* in the case of Erbb2-like^Ex^ and *ESR1* in the case of Neu^Ex^). Three hypotheses that could explain this finding are: 1) the cell type of origin of the tumor (but not a genetic driver) is the same across species and this is the major linking phenotype; 2) additional unknown genetic driver(s) are responsible for the common phenotype across species; or 3) some combination of hypothesis 1 and 2. We favor the common cell type of origin hypothesis, but additional experiments like lineage tracing will be required to unequivocally determine this.

Related to this, there are at least two confounding features within our dataset that should also be considered when interpreting these results. First, most of the oncogene-driven mouse models analyzed here used either the MMTV or WAP promoter in their design. If the activity of these promoters varies as a function of specific mammary cell types, such as luminal versus myoepithelial cells, then only those cells that naturally use these promoters would ever give rise to a tumor in these models; we note that most of the MMTV or WAP driven tumors were luminal. Second, similar complications potentially exist with regards to mouse strain. Varying the background genetics in which a model is designed can influence tumor phenotype, and thus classification. Unfortunately, our dataset is underpowered to adequately address these two confounding features, but future experiments/models could be designed to address these questions.

While some of the mouse classes were identified as good counterparts for specific human subtypes, many were not. There are several possibilities to explain this lack of association. The first is that these classes are specific to murine mammary carcinomas and do not have a matching counterpart in humans. The second might be that these murine classes model rare phenotypes that exist in only a small subset of human breast cancer patients, and that these rare human subtypes were not present in the datasets used here. Similarly, more mouse tumors for classes with small numbers may be required to increase statistical power to detect an association; for example, we hypothesize this to be the case for the PyMT^Ex^ class. The third possibility is that these novel murine classes share phenotypes with multiple human subtypes, and thus may never be classified as being similar to a single human subtype. Some murine tumor features were shared across multiple human subtypes (for example, Myc^Ex^ with human basal-like and luminal B), which our presented analysis is more likely to undervalue. While this study provides a framework for identifying GEMMs that could be useful for preclinical drug testing, the simultaneous analysis of 27 mouse models restricted our *trans*-species comparisons to only expression-based analyses. The scope of our future work will focus on using models selected based upon these data for preclinical therapeutic testing to better determine the translational utility of these GEMMs. These experiments are already underway and producing promising results using the TgMMTV-*Neu*, TgC3(1)-*Tag*, and claudin-low T11 models [48,51-53]. For example, in Roberts *et al.*[51], we showed that the CyclinD1 dependent TgMMTV-Neu tumors are sensitive to a CDK4/6 inhibitor, while the basal-like TgC3(1)-*Tag* tumors were not; these studies are consistent with findings coming from human clinical trials of luminal/ER + breast cancers, which were generally noted to be sensitive to a CDK4/6 inhibitor [54]. Similarly, a *trans*-species genetic screen by Bennett *et al.*[53] identified two ribonucleotide reductase genes (*RRM1* and *RRM2*) and a checkpoint kinase (*CHK1*) as potential targets for triple-negative breast cancer patients, which they validated in both species with drug treatment experiments using TgC3(1)-*Tag* and human xenograft tumors.

Lastly, the data presented in this study may provide an explanation for a recent paper that concluded that murine models are not helpful for studying acute human inflammatory disease [55]. Their conclusion was drawn from a comparison using a single mouse strain/model (that is, C57BL6) versus a large number of humans. Based on the data presented here, we predict that multiple mouse strains/models would need to be tested before such a conclusion could be made. To improve preclinical study designs using mouse models for any disease, it is our recommendation that the following steps be used as guidelines: 1) select/create multiple mouse models for comparative analysis to humans; 2) classify the phenotype(s) of each model with a specific focus on the degree of intramodel ‘heterogeneity’; and 3) objectively compare each model to the human disease state to identify the possible *trans*-species counterparts. With this approach, it is likely that some strains/models might be rejected as not mimicking the human disease state, while others may, and it is those that do that are the most valuable for preclinical testing. We suggest that the use of this approach will increase the predictive nature of preclinical studies in mice.

## Conclusion

We consolidate 27 murine models of breast carcinoma into the largest comprehensive genomic dataset to date, and we provide a detailed characterization of each to better understand how these GEMMs recapitulate phenotypes of the human subtypes. The data presented here provide insight into the molecular pathways involved in specific breast cancer subtypes and should serve as a useful resource when designing preclinical studies and interpreting their results.

## Materials and methods

### Gene expression microarrays

A murine tumor dataset of 385 DNA gene expression microarrays from 27 GEMMs of mammary carcinoma was compiled (Table 1A; Table S1 in Additional file 1). Of these, 275 samples were obtained from multiple previous publications (Gene Expression Omnibus accession numbers GSE3165, GSE8516, GSE9343, GSE14457, GSE15263, GSE17916, and GSE27101). The other 110 microarray samples (GSE42640) represent newly obtained tumor samples from multiple participating investigators using methods approved by international animal husbandry guidelines. Total RNA was purified from 20 to 30 mg of mouse mammary tumor using Qiagen’s (Valencia, CA USA) RNeasy Mini Kit following the manufacture’s protocols. RNA quantity and quality were determined using the Nanodrop spectrophotometer and Agilent Bioanalyzer, respectively. Total RNA was reverse transcribed and labeled with cyanine-5 (Cy5) dye for experimental samples and cyanine-3 (Cy3) dye for mouse reference samples [31] using the Agilent Low RNA Input Fluorescent Linear Amplification Kit. Equal quantities of labeled mouse reference RNA and tumor RNA were co-hybridized overnight to Agilent microarrays, washed, scanned and signal intensities were determined.

All tumor samples were co-hybridized to one of three Agilent Technology gene expression microarray types: 22 K, 4X44K, or 4X180K (Figure 1). Two ‘homogeneous expression’ murine models [31], namely TgMMTV-*Neu* and TgC3(I)-*Tag*, were analyzed on all three array types. Therefore, we used both of these models to normalize expression between microarray types [32]. Ten microarrays (five TgMMTV-*Neu* and five TgC3(I)-*Tag*) from each array type were used for normalization (30 microarrays total). All microarray data were independently extracted from the UNC Microarray Database for each array type as log_2_ Cy5/Cy3 ratios, filtering for probes with Lowess normalized intensity values greater than 10 in both channels and for probes with data on greater than 70% of the microarrays [31,34]. Before normalization, each data set was imputed (via the 10 nearest neighbor gene values) and then reduced to the probes that were present on all three array type datasets (11,690 probes, 11,167 genes). Using the 10 normalization arrays per 3 array platforms, the median expression value was calculated for each probe, on each array type, and a normalization factor was applied independently to each probe so the median was the same for each array type. Probe expression values were ‘median centered’ to obtain the final normalized dataset. A principle component analysis was performed to verify the normalization.

### Murine intrinsic genes and subtypes

After removing technical replicates, the dataset was filtered to probes with at least three observations with an absolute log_2_ expression value >3 using Gene Cluster 3.0 [56], which included 908 probes (899 genes). Hierarchical clustering was performed with this unsupervised probe list using centroid linkage and was viewed with Java Treeview v1.1.5r2 [57]. Potential ‘intrinsic groups’ of murine samples were defined as any set of samples/arrays within this hierarchical cluster that had a Pearson correlation value of 0.65 or greater [31]. Using these defined groups (42 total), an ‘intrinsic gene list’ of 1,855 probes (1,841 genes) was identified with Intrinsic Gene Identifier v1.0 (Max Diehn/Stanford University) by using a cutoff of one standard deviation below the mean intrinsic gene value [31] (Table S2 in Additional file 1).

To identify significant murine ‘intrinsic subtypes’, the 385 sample dataset was clustered again using the 1,855 intrinsic probe list and SigClust [37] was used to identify groups of samples with a significant association to one another (*P* < 0.01) [32]. GEMM classes were defined as having at least five tumors and a SigClust *P*-value ≤0.01, yielding 17 classes. Class-specific probes/genes were determined using a two class (class X versus all other samples) SAM analysis (v3.11) [34,58] (Tables S4 to S20 in Additional file 1).

### Human and mouse intrinsic gene co-cluster

Prior to combining the two datasets, probes corresponding to orthologous gene IDs (as determined by the Mouse Genome Informatics of the Jackson Laboratory) were averaged for both the mouse and UNC308 human datasets. Using only orthologous genes found in both datasets (8,034 genes), each tumor and gene was standardized to have an average expression of zero and a standard deviation of one (N(0,1)) separately for each species. Then, the datasets were merged and each gene was median centered to obtain the final, normalized combined dataset. A merged intrinsic gene list was created by combining the 1,841 mouse intrinsic genes defined here and the 1,918 human intrinsic genes from Parker *et al.*[41] (3,310 unique genes in the combined gene set). An intrinsic gene set hierarchical co-cluster was performed using centroid linkage in Gene Cluster 3.0.

### Comparison of murine and human expression subtypes

To identify possible commonalities between mouse classes and the human intrinsic subtypes of breast cancer [34,41], we used the GSA R package v1.03 [42] and R v2.12.2. Human subtype-specific gene lists were derived for each subtype with a two class (subtype X versus all other samples) SAM analysis independently for all of the unique primary tumor samples from Prat *et al.*[34] (referred to as the UNC308 dataset), from Harrell *et al.*[43] (Combined855 dataset), and from TCGA 2012 (TCGA547 dataset) [39] (Tables S21 to S26 in Additional file 1). Human subtype-specific genes were classified as being highly expressed in the subtype of interest and having a SAM FDR of 0%. Murine classes were then analyzed for significant overlap with each dataset’s human subtype-specific gene sets using GSA. Significant overlap was defined as having *P* ≤ 0.05 and FDR ≤0.1 to control for multiple comparisons [42]. These same methods were used to identify significant overlap between our 17 newly derived murine classes and the 10 previously defined GEMM classes from Herschkowitz *et al.*[31], noting that all 122 arrays used for the Herschkowitz *et al.* study were also present within the 385 sample dataset used here (Figure S3 in Additional file 2).

### Conserved pathway gene signatures

Only genes that were found in both the human and murine datasets were considered for gene-signature analysis in order to eliminate the influence of genes found in only one dataset. Prior to calculating gene-signature values, the human and murine datasets were separately collapsed by averaging rows corresponding to the same gene symbol. Median expression values were calculated for 963 publicly available pathway-based gene signatures using methods described in Fan *et al.*[59,60] (Table S3 in Additional file 1). A two class SAM (class or subtype X versus all other samples) was used to identify pathway signatures enriched in murine and human classes/subtypes, which were defined as being upregulated with a FDR of 0% (Tables S4 to S26 in Additional file 1).

## Abbreviations

Cy3: Cyanine-3; Cy5: Cyanine-5; ER: Estrogen receptor; FDR: False discovery rate; GEMM: Genetically engineered mouse model; GSA: Gene set analysis; SAM: Significance Analysis of Microarrays.

## Competing interests

CMP is an equity stock holder of BioClassifier LLC and University Genomics, and has filed a patent on the PAM50 subtyping assay.

## Authors’ contributions

Conception and design: ADP and CMP. Tumor collection: JIH, JU, JRA, MIT, MB, and SEE. Acquisition of data: ADP, JIH, JU, and JCH. Analysis and interpretation of data: ADP, JIH, JU, JCH, BTS, GMW, JMR, and CMP. Writing of manuscript: ADP and CMP. All authors read and approved the final manuscript.

## Supplementary Material

Additional file 1: Tables S1 to S26 A table of contents is listed on the first worksheet that describes the information presented in Tables S1 to S26. Click here for file

Additional file 2: Figures S1 to S3 Figure S1: enlarges the cluster dendrogram from Figure 2B, showing the clustering location and expression class for each individual tumor in the mouse dataset. Figure S2: clustering location for tumors of a given model from Figure 2B. Figure S3: gene set analysis results comparing the 10 murine classes from Herschkowitz *et al.*[31] and the 17 murine classes defined here. Click here for file

## Data access

Microarray data have been deposited in the Gene Expression Omnibus (GEO) under accession number GSE42640.
