# Supplementary material for: Transcriptomic classification of genetically engineered mouse models of breast cancer identifies human subtype counterparts
Source: Genome Biol. 2013 Nov 12;14(11):R125. doi: 10.1186/gb-2013-14-11-r125 (PMC4053990; doi:10.1186/gb-2013-14-11-r125)
Supplement: Additional file 2: Figures S1 to S3 — Figure S1: enlarges the cluster dendrogram from Figure 2B, showing the clustering location and expression class for each individual tumor in the mouse dataset. Figure S2: clustering location for tumors of a given model from Figure 2B. Figure S3: gene set analysis results comparing the 10 murine classes from Herschkowitz et al.[31] and the 17 murine classes defined here. [file gb-2013-14-11-r125-S2.pdf]

Supplemental Figure 1

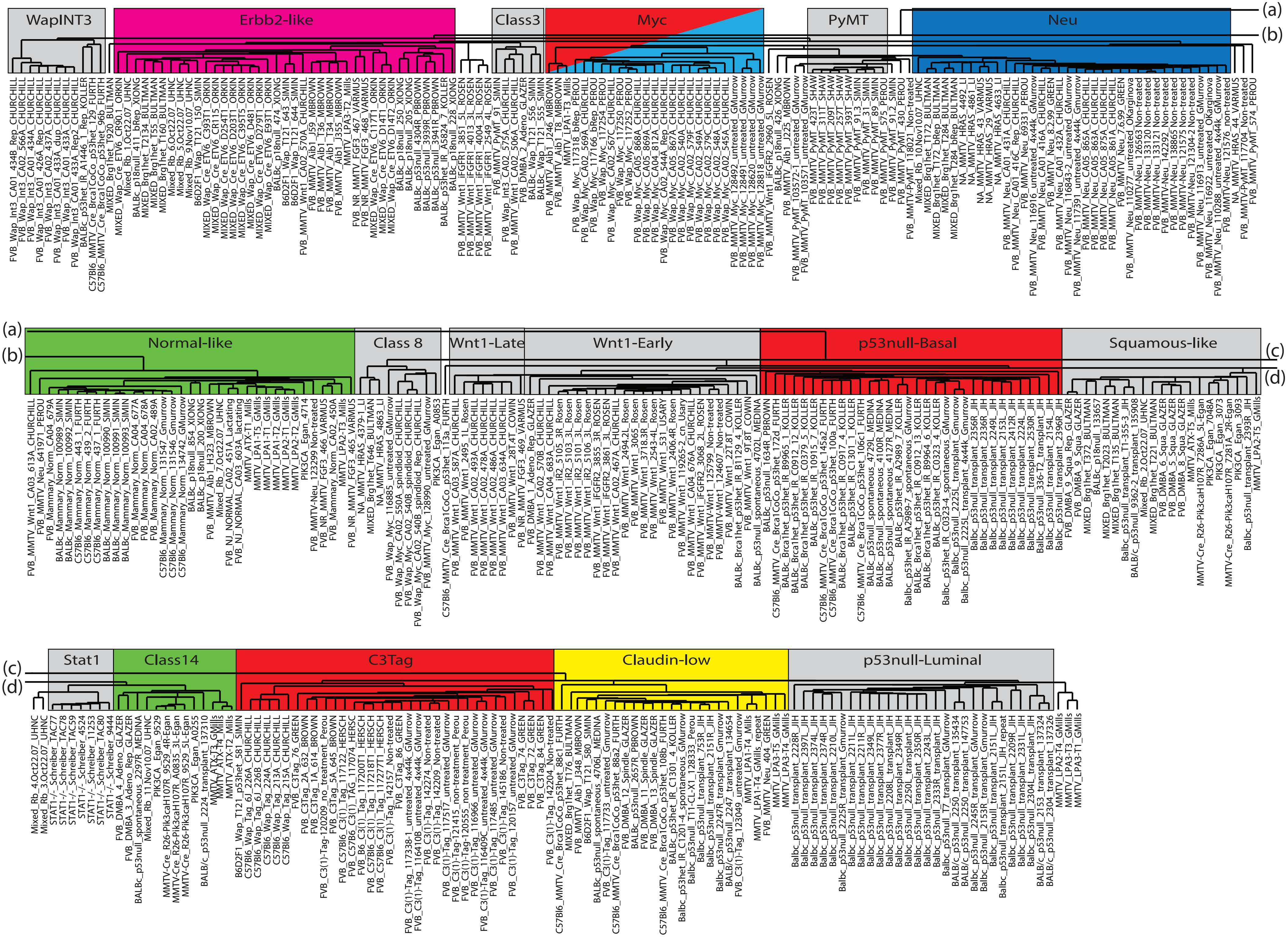

Supplemental Figure 2

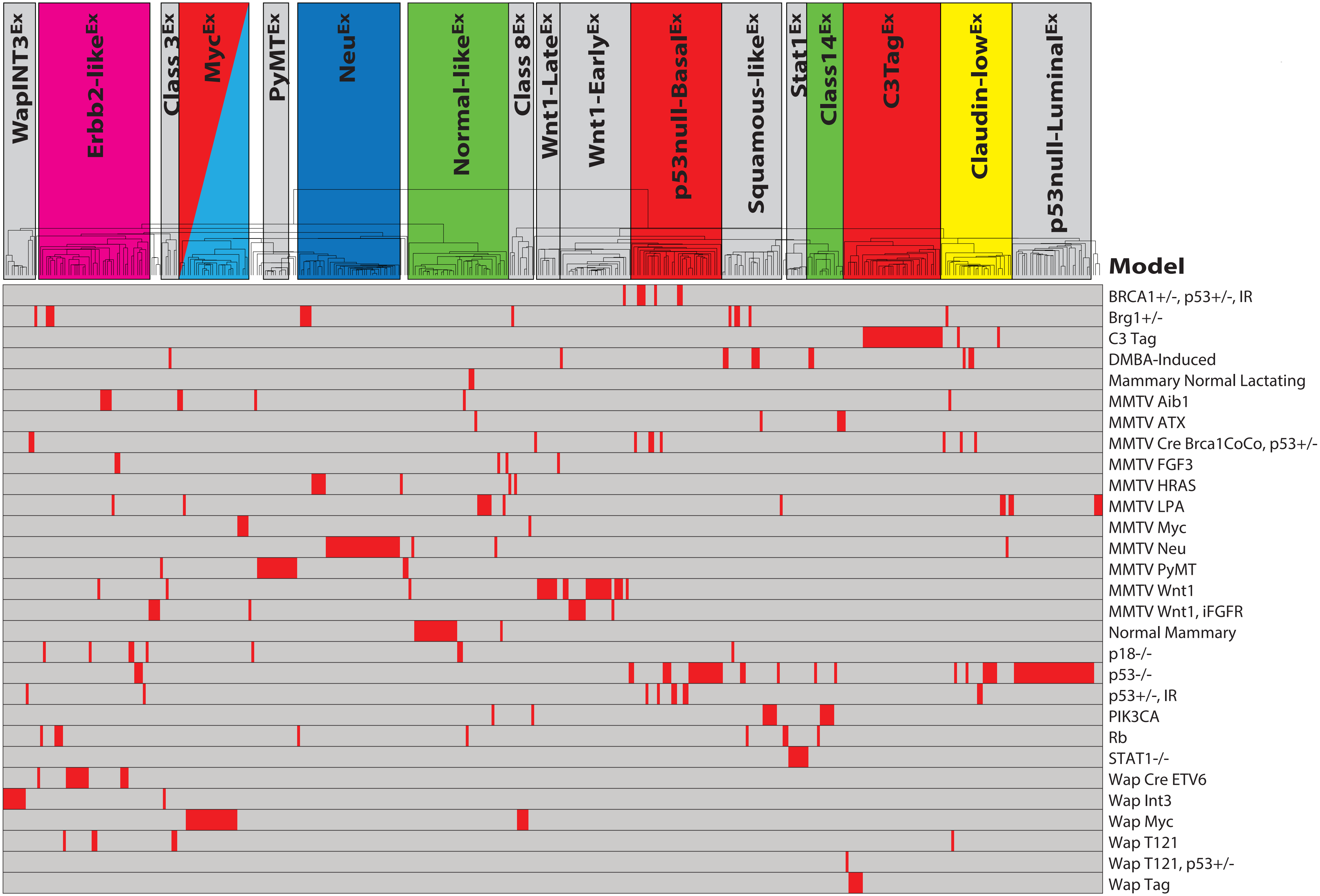

Supplemental Figure 3

Class	I-Normal	II-Claudin-low	III-DMBAwnt	IV-BRCAwnt	V-p53null	VI-NeuPyMT	VII-Myc	VIII-WapINT3	IX-WapTag	X-C3Tag	Predicted Herschkowitz Class
	Herschkowitz et al 2007 Class										
**WapINT3[Ex]**	-	-	-	-	-	-	0.324	**<1.0e-4**	0.352	0.405	WapINT3
**Erbb2-like[Ex]**	-	-	-	0.444	0.059	0.169	0.234	-	**0.046**	-	
**Class3[Ex]**	0.275	0.457	0.162	-	-	-	0.288	0.131	-	-	
**Myc[Ex]**	-	-	-	-	-	-	**<1.0e-4**	-	-	-	Myc
**PyMT[Ex]**	-	-	-	-	-	**0.009**	0.092	-	0.464	-	NeuPyMT
**Neu[Ex]**	0.322	-	-	-	-	**<1.0e-4**	-	0.363	-	-	NeuPyMT
**Normal-like[Ex]**	**<1.0e-4**	0.286	0.289	-	-	0.308	-	0.349	-	-	Normal
**Class8[Ex]**	0.375	0.104	-	-	-	0.386	0.323	0.337	-	-	
**Wnt1-Late[Ex]**	-	-	**<1.0e-4**	0.275	-	-	0.357	**0.042**	-	-	DMBAwnt
**Wnt1-Early[Ex]**	-	-	**0.020**	**0.005**	-	0.475	0.344	0.221	-	0.410	BRCA/DMBAwnt
**p53null-Basal[Ex]**	-	0.275	0.089	**<1.0e-4**	0.068	-	-	-	0.339	0.382	BRCAwnt
**Squamous-like[Ex]**	0.327	0.104	**0.003**	0.325	-	-	-	0.353	-	-	DMBAwnt
**Stat1[Ex]**	0.382	0.232	0.164	0.421	0.464	-	-	-	0.431	0.337	
**Class14[Ex]**	0.366	0.376	**0.001**	0.278	-	-	-	-	-	-	DMBAwnt
**C3Tag[Ex]**	-	-	-	-	0.454	-	-	-	**0.003**	**<1.0e-4**	C3/WapTag
**Claudinlow[Ex]**	-	**<1.0e-4**	-	0.483	0.330	-	-	-	0.464	-	Claudin-low
**p53null-Luminal[Ex]**	-	0.286	-	0.298	**0.001**	-	0.239	-	0.125	0.339	p53null
